# Replication and Predictive Value of SNPs Associated with Melanoma and Pigmentation Traits in a Southern European Case-Control Study

**DOI:** 10.1371/journal.pone.0055712

**Published:** 2013-02-05

**Authors:** Irene Stefanaki, Orestis A. Panagiotou, Elisavet Kodela, Helen Gogas, Katerina P. Kypreou, Foteini Chatzinasiou, Vasiliki Nikolaou, Michaela Plaka, Iro Kalfa, Christina Antoniou, John P. A. Ioannidis, Evangelos Evangelou, Alexander J. Stratigos

**Affiliations:** 1 Department of Dermatology, University of Athens Medical School, Andreas Sygros Hospital, Athens, Greece; 2 Department of Hygiene and Epidemiology, University of Ioannina School of Medicine, Ioannina, Greece; 3 Department of Internal Medicine, University of Athens, Laikon Hospital, Athens, Greece; 4 Blood Donation Unit, Laikon Hospital, Athens, Greece; 5 Stanford Prevention Research Center, Department of Medicine and Department of Health Research and Policy, Stanford University School of Medicine, and Department of Statistics, Stanford University School of Humanities and Sciences, Stanford, California, United States of America; 6 Department of Twin Research and Genetic Epidemiology, King's College London, London, United Kingdom; King's College London, United Kingdom

## Abstract

**Background:**

Genetic association studies have revealed numerous polymorphisms conferring susceptibility to melanoma. We aimed to replicate previously discovered melanoma-associated single-nucleotide polymorphisms (SNPs) in a Greek case-control population, and examine their predictive value.

**Methods:**

Based on a field synopsis of genetic variants of melanoma (MelGene), we genotyped 284 patients and 284 controls at 34 melanoma-associated SNPs of which 19 derived from GWAS. We tested each one of the 33 SNPs passing quality control for association with melanoma both with and without accounting for the presence of well-established phenotypic risk factors. We compared the risk allele frequencies between the Greek population and the HapMap CEU sample. Finally, we evaluated the predictive ability of the replicated SNPs.

**Results:**

Risk allele frequencies were significantly lower compared to the HapMap CEU for eight SNPs (rs16891982 – *SLC45A2*, rs12203592 – *IRF4*, rs258322 – *CDK10*, rs1805007 – *MC1R*, rs1805008 - *MC1R*, rs910873 - *PIGU*, rs17305573- *PIGU*, and rs1885120 - *MTAP*) and higher for one SNP (rs6001027 – *PLA2G6*) indicating a different profile of genetic susceptibility in the studied population. Previously identified effect estimates modestly correlated with those found in our population (r = 0.72, P<0.0001). The strongest associations were observed for rs401681-T in *CLPTM1L* (odds ratio [OR] 1.60, 95% CI 1.22–2.10; P = 0.001), rs16891982-C in *SCL45A2* (OR 0.51, 95% CI 0.34–0.76; P = 0.001), and rs1805007-T in *MC1R* (OR 4.38, 95% CI 2.03–9.43; P = 2×10^−5^). Nominally statistically significant associations were seen also for another 5 variants (rs258322-T in *CDK10*, rs1805005-T in *MC1R*, rs1885120-C in *MYH7B*, rs2218220-T in *MTAP* and rs4911442-G in the *ASIP* region). The addition of all SNPs with nominal significance to a clinical non-genetic model did not substantially improve melanoma risk prediction (AUC for clinical model 83.3% versus 83.9%, p = 0.66).

**Conclusion:**

Overall, our study has validated genetic variants that are likely to contribute to melanoma susceptibility in the Greek population.

## Introduction

Plethora of studies has shown that ultra-violet (UV) light exposure and certain phenotypic traits, i.e. red or blonde hair, light-colored eyes, fair skin complexion, and prominent mole pattern are major risk factors for the development of cutaneous melanoma (CM) [1]–[6]. A strong genetic background has been supported by twin studies showing a 55% contribution of genetic effects in melanoma variation liability [7].

High-penetrance germline mutations in *CDKN2A* and *CDK4* genes are rare (0.2–1.2%) in sporadic CM, but they are encountered in approximately 5% of families with only two members with CM, and in 30–40% of families with 3 or more affected members [8]–[10]. The advent of high-throughput genotyping technologies and their utilization in population-based studies has discovered a considerable number of rare and common genetic variants at different genetic loci associated with melanoma. The most prevalent low penetrance locus is the melanocortin 1 receptor gene (*MC1R*), whose variants have been associated both with melanoma [11]–[18] as well as with related traits [11], [19]–[21]. Apart from *MC1R*, a significant number of low penetrance genes involved in various cellular pathways, such as pigmentation, cell cycle control, DNA repair, oxidation stress, apoptosis, senescence and melanocyte differentiation and migration have been implicated in melanoma susceptibility [22]. A detailed synopsis and meta-analysis of reported melanoma-associated variants is available in MelGene, an on-line database (http://www.melgene.org) [23]. In addition to common variants, a rare germline variant in *MITF* (rs14917956 – E318K) that alters *MITF* transcriptional activity was recently found to be associated with melanoma and renal cell cancer [24]–[25].

Most genetic association studies on CM have been performed in populations with fair skin and, hence, the effect of melanoma-associated variants in relatively darker skin populations residing in areas of higher ambient UV-exposure is less well known. Being a southern European country, Greece is characterized by a high degree of sun exposure year-round, a population of relatively darker skin complexion compared to northern European countries and the lowest incidence of melanoma (4–5 per 100,000 person-years) among European countries [26]–[28]. Mutational analyses performed by our group in Greek patients with sporadic and genetically enriched melanoma, found a higher prevalence of *CDKN2A/CDK4* mutations than previously reported, suggesting a more prominent role of genetic susceptibility to melanoma in regions with a relatively low incidence of melanoma [28]–[29]. In the present study, we sought to replicate the most prominent results of MelGene and other findings from genome-wide association studies (GWAS) in a Greek case-control study. Our research replicates a number of variants that are associated with melanoma risk in the Greek population and the relevant pathogenetic pathways that are involved; it also highlights differences in risk allele frequencies among the Greek population and the HapMap European sample concerning mainly pigmentation-related risk loci. Finally, it provides insights about the predictive value of identified genetic risk factors compared to well-established clinical ones.

## Materials and Methods

### Study Population

The study population consisted of Greek melanoma cases and control subjects, above 18 years of age. The case sample consisted of patients diagnosed with non-familial, histologically confirmed invasive melanoma at A. Sygros Hospital, a large referral center of melanoma in Athens, and participating melanoma centers, from 2003 to 2009. The control sample included blood donors from a blood donation center and individuals with minor skin diseases attending A. Sygros Hospital. Controls were matched 1∶1 on age (+/−2 years) and gender to the cases. Individuals with a history of melanoma, other types of skin cancer, or any non-dermatological malignancy were excluded from the control arm of the study.

Each subject was interviewed and examined by a dermatologist or trained physician and information was retrieved on demographic variables (age, sex), pigmentation traits (eye, hair, and skin color), phototype, and sun exposure variables (sunburns, tanning). The Declaration of Helsinki protocols were followed and the Scientific and Ethics Committee of Andreas Sygros Hospital has reviewed and approved the research protocol; all participating individuals gave written informed consent.

### SNP selection and Genotyping

All variants included in this study were selected from the last update of the MelGene field synopsis (October 2011), a large on-line database that was created with the purpose of comprehensively collecting and meta-analyzing all published genetic associations of melanoma (http://www.melgene.org) [23]. More specifically, the 34 selected variants from MelGene were distinguished in two groups: 1) all variants associated with melanoma at a level of p<0.05 following meta-analysis of relevant data from at least 3 independent case-control datasets (28 variants) and 2) additional biologically plausible variants representing potential causal pathways and selected from GWAS (3 variants) and candidate gene studies (3 variants) with genome-wide (p<10^−7^) or nominally significant (p<0.05) associations. These variants were also included in MelGene but not necessarily meta-analyzed due to insufficient number of available datasets. In all, of the 34 variants, 19 had reached genome-wide significance in a previous GWAS or in MelGene, and the other 15 had not.

### DNA isolation, Genotyping and Quality control

Genomic DNA was isolated from peripheral blood using the QIAamp DNA blood mini kit (Qiagen). DNA concentration was quantified in samples prior to genotyping by using Quant-iT dsDNA HS Assay kit (Invitrogen). The concentration of the DNA was adjusted to 5 ng/µl.

A total of 50 ng from each DNA sample were used to genotype the selected 34 SNPs using the Sequenom iPLEX assay (Sequenom, Hamburg, Germany). Allele detection in this assay was performed using matrix-assisted laser desorption/ionization –time-of-flight mass spectrometry [30].

Our quality control criteria included the inclusion of SNPs with a genotype call rate of 95% or higher, as well as SNPs showing no deviation from Hardy-Weinberg equilibrium (HWE) in the controls using a chi-squared test (P>0.05).

### Statistical Analysis

We examined the association of each SNP with CM by performing conditional logistic regression analyses assuming a multiplicative model of inheritance considering the minor allele as the reference allele. To control for the effect of the other covariates/risk factors on CM in the Greek population, each SNP was subsequently incorporated into multivariable logistic regression models using a stepwise variable selection approach. The covariates considered were eye color (light: blue, green/gray and light brown or dark: dark brown and black), hair color (light: blond/red and light brown or dark: dark brown and black), skin color (light: fair/pale and light brown or dark: dark brown), phototype (type I, II, III or IV, according to the Fitzpatrick scale), tanning ability (burn, minimal tan, burn then tan or deep tan), and sunburn (presence or absence). We estimated odds ratios and 95% confidence intervals (95% CI) for all models. Missing values for any of the non-genetic risk factors were imputed using multiple imputation methods. Variables where all the required information was available were used for the construction of the models for the estimation of the imputed missing values.

Additionally, we estimated the correlation of risk allele frequencies between the HapMap CEU sample and the Greek population across all the evaluated SNPs. Moreover, we estimated the correlation of the effect sizes found in the Greek population with those found previously in the original publications or MelGene dependent on the source of SNP selection. We examined whether the direction of the effect estimates was in the same or in opposite directions.

For the sample size of our study, we estimated the power *G_i_* to detect each of the previously described effects at α = 0.05 level given the observed minor allele frequency in the Greek studied population assuming a multiplicative (per-allele) genetic model. We used the QUANTO software (http://hydra.usc.edu/gxe, accessed 30 September 2012). The sum of the power estimates corresponds to the number of variants that would be expected to replicate. Subsequently we calculated the binomial test for the expected vs. the replicated variants across all evaluated SNPs.

Finally, we created receiver operating characteristic (ROC) curves to assess the predictive ability of the CM-associated SNPs. We considered 3 models including, respectively, the phenotypic traits alone (model 1); the phenotypic traits along with the SNPs that remain statistically significant after Bonferroni correction (model 2); and the phenotypic traits along with all nominally statistically significant SNPs (model 3). In order to assess the validity of our models, we used k-fold cross-validation with k = 2 splits and 1,000 replications.

All statistical analyses were performed in STATA version 11.2 (College Station, TX, USA). All P-values are two-tailed.

## Results

Our sample included 284 patients with CM matched on age and sex to 284 controls; of those, 270 (48%) were men. Median age was 44 years (range 18–85) for patients and 42 years (range 18–81) for controls. Demographics and phenotypic traits are shown in Table S1. Missing values in phenotypic characteristics were due to the fact that blood samples and questionnaires in one participating center were collected in the early phase of this study, and the corresponding individuals could not be found in order to retrieve these data. A total of 34 variants were selected for genotype analysis (Table 1). All of them were successfully genotyped with call rates of 95% or above. Deviation from HWE in the control population was noticed for one single-nucleotide polymorphism (SNP) (rs4636294), which was subsequently excluded from further statistical analyses.

**Table 1:** Summary results for SNPs selected for replication in the present Greek case-control study.

SNP	Minor/Major alleles in the Greek sample	Gene Locus	Position/Function	MAF (95% CI) in the Greek sample	MAF in the reference source^1^	OR in the Greek sample (95% CI)	OR (95% CI) in the reference source^1^	GWAS significant	Reference source^1^
rs16891982	C/G	*SLC45A2*	exon	0.14 (0.11–0.17)	0.068	0.51 (0.34–0.76)	0.40 (0.33–0.47)	Yes	Melgene
rs401681	T/C	*CLPTM1L*	intron	0.40 (0.36–0.44)	0.450	1.60 (1.22–2.10)	1.15 (1.08–1.22)	Yes	Melgene
rs12203592	T/C	*IRF4*	intron	0.04 (0.03–0.07)	0.2259	1.08 (0.62–1.89)	1.04 (0.99–1.08)	No	Candidate gene study [44]
rs7023329	G/A	*MTAP*	intron	0.40 (0.36–0.44)	0.497	0.95 (0.73–1.25)	0.84 (0.80–0.89)	Yes	Melgene
rs11515	G/C	*CDKN2A*	3′ UTR	0.18 (0.15–0.21)	0.127	0.97 (0.70–1.35)	1.15 (1.01–1.32)	No	Melgene
rs3088440	A/G	*CDKN2A*	3′ UTR	0.07 (0.05–0.09)	0.077	1.41 (0.89–2.21)	1.25 (1.03–1.51)	No	Melgene
rs4636294^2^	G/A	*MTAP region*	intergenic	0.41 (0.36–0.45)	0.485	N/A	0.82 (0.75–0.90)	Yes	Melgene
rs1335510	G/T	*MTAP region*	intergenic	0.31 (0.27–0.35)	0.417	0.86 (0.64–1.15)	0.83 (0.78–0.89)	Yes	Melgene
rs2218220	T/C	*MTAP region*	intergenic	0.41 (0.37–0.46)	0.487	0.74 (0.56–0.97)	0.84 (0.80–0.89)	Yes	Melgene
rs10757257	A/G	*MTAP*	intron	0.31 (0.27–0.35)	0.415	0.87 (0.65–1.16)	0.81 (0.76–0.86)	Yes	Melgene
rs1011970	T/G	*CDKN2B*	5′ UTR	0.16 (0.13–0.20)	NA	1.27 (0.91–1.78)	NA	No	GWAS [49]
rs1408799	T/C	*TYRP-1region*	intergenic	0.32 (0.28–0.36)	0.258	1.16 (0.87–1.54)	0.86 (0.80–0.93)	No	Melgene
rs1042602	A/C	*TYR*	exon	0.46 (0.42–0.51)	0.316	1.11 (0.85–1.46)	0.95 (0.90–0.99)	No	Melgene
rs1126809	A/G	*TYR*	exon	0.20 (0.16–0.23)	0.295	1.19 (0.88–1.62)	1.22 (1.14–1.31)	Yes	Melgene
rs1393350	A/G	*TYR*	intron	0.19 (0.16–0.23)	0.269	1.16 (0.85–1.58)	1.27 (1.16–1.39)	Yes	Melgene
rs1544410	A/G	*VDR*	intron	0.42 (0.38–0.46)	0.400	0.95 (0.72–1.24)	0.89 (0.82–0.97)	No	Melgene
rs1800407	A/G	*OCA2*	exon	0.06 (0.04–0.08)	0.070	1.32 (0.81–2.16)	1.4 (1.07–1.82)	No	Melgene
rs258322	T/C	*CDK10*	intron	0.05 (0.03–0.07)	0.095	2.26 (1.32–3.88)	1.66 (1.48–1.86)	Yes	Melgene
rs1805005	T/G	*MC1R*	exon	0.13 (0.11–0.16)	0.114	1.59 (1.09–2.32)	1.13 (1.02–1.26)	No	Melgene
rs1805007	T/C	*MC1R*	exon	0.02 (0.01–0.03)	0.078	4.38 (2.03–9.43)	1.83 (1.56–2.15)	Yes	Melgene
rs1805008	T/C	*MC1R*	exon	0.02 (0.01–0.04)	0.098	1.64 (0.85–3.19)	1.54 (1.33–1.79)	Yes	Melgene
rs1805009^3^	C/G	*MC1R*	exon	0	0.015	N/A	1.89 (1.51–2.38)	Yes	Melgene
rs1805006	A/C	*MC1R*	exon	0.005 (0.001–.02)	0.009	0.33 (0.03–3.20)	1.47 (1.18–1.83)	No	Melgene
rs11547464^3^	T/G	*MC1R*	exon	0	0.010	N/A	1.67 (1.26–2.21)	No	Melgene
rs4785763	A/C	*AFG3L1*	3′UTR	0.28 (0.24–0.32)	0.328	1.29 (0.97–1.72)	1.36 (1.27–1.45)	Yes	Melgene
rs6058017	G/A	*ASIP*	3′ UTR	0.14 (0.12–0.18)	0.089	1.10 (0.78–1.54)	0.89 (0.81–0.99)	No	Melgene
rs4911414	T/G	*ASIP region*	intergenic	0.24 (0.21–0.28)	0.31	0.86 (0.64–1.16)	1.21 (0.96–1.51)	No	Candidate gene study [50]
rs1015362	A/G	*ASIP region*	intergenic	0.27 (0.24–0.31)	0.27	0.91 (0.68–1.21)	0.89 (0.69–1.13)	No	Candidate gene study [50]
rs910873	A/G	*PIGU*	intron	0.02 (0.01–0.03)	0.076	2.11 (0.96–4.67)	1.52 (1.36–1.70)	Yes	Melgene
rs17305573	C/T	PIGU	intron	0.02 (0.01–0.03)	0.09	2.11 (0.96–4.67)	1.53 (1.25–1.87)	No	GWAS [49]
rs1885120	C/G	*MYH7B*	intron	0.02 (0.01–0.03)	0.073	2.22 (1.01–4.88)	1.59 (1.41–1.79)	Yes	Melgene
rs4911442	G/A	*NCOA6*	intron	0.04 (0.03–0.07)	0.15	1.79 (1.02–3.14)	1.2 (0.99–1.46)	Yes	GWAS [51]
rs2284063	G/A	*PLA2G6*	intron	0.36 (0.32–0.40)	0.366	1.12 (0.87–1.43)	0.85 (0.75–0.96)	Yes	Melgene
rs6001027	T/C	*PLA2G6*	intron	0.30 (0.27–0.34)	0.363	0.88 (0.67–1.17)	0.86 (0.77–0.96)	Yes	Melgene

1 Reference source = Melgene: nominal association with melanoma after meta-analysis of data for this variant derived from at least 3 datasets (MelGene is an online database of all reported genetic associations of melanoma which includes a systematic meta-analysis of melanoma-associated variants from published datasets and grading of this associations for strength of epidemiogical evidence) [23], or data derived from original study (variants not meta-analyzed in Melgene).

2 Showed deviation from HWE, and was therefore not included in the analyses: N/A for MAF & OR in the Greek sample.

3 All individuals were homozygous for the major allele.

Abbreviations: MAF, minor allele frequency; CI, confidence interval; OR, odds ratio.

From the selected variants, four SNPs are found in the 3′-UTRs and one in the 5′- UTR of the respective gene loci; 13 are located in introns; and 10 are within exons. The remaining 6 variants are found in intergenic positions. We found evidence for strong pair-wise LD (r^2^>0.85) between rs2218220 and rs4636294 (r^2^ = 0.95), which deviated from HWE; rs10757257 and rs1335510 (r^2^ = 0.96); rs1393350 and rs1126809 (r^2^ = 0.94); and rs1885120, rs910873 and rs17305573 (r^2^ = 0.90). For the remaining, moderate LD was observed (r^2^<0.60).

### Association of variants with CM risk

Table 1 shows the 33 analyzed SNPs, their effect sizes, minor and major alleles and the corresponding frequencies in the Greek population. All alleles identified as minor in the Greek population were also minor alleles in the CEU HapMap sample with one exception (rs6001027 whose minor allele was T in the Greek population but C in HapMap CEU).

**Figure 1:**
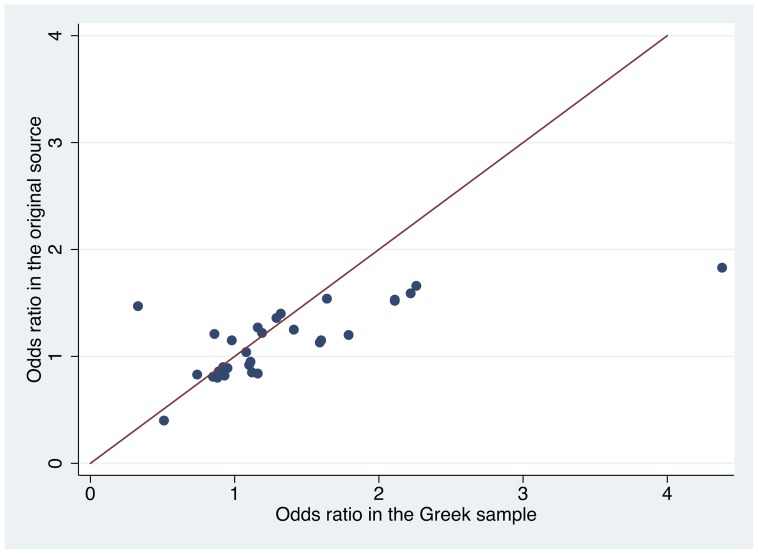
Correlation of effect sizes. Correlation of the effect sizes found in the Greek population and those described in the original publication or MelGene..Not shown are: rs4636294 (excluded from analyses because of HWE deviation); rs1011970 because OR was not available in the original publication and/or MelGene; rs1805009 and rs11547464 because all subjects were homozygous for the major allleles.

Figure 1 shows the correlation between the ORs identified for the 33 eligible SNPs in the Greek population and in the original source where these were selected. We noticed overall modestly high correlation of the respective effect estimates (r = 0.72, P<0.0001). No differences in ORs between the Greek population and the original source were beyond chance (i.e. 95% CI between the two populations showed overlap for each SNP). Overall, no nominally significant difference in ORs was noticed across all SNPs in the two populations (P = 0.411 for Mann-Whitney U).

When limited to SNPs that had previously reached genome-wide significance in either Melgene or a previous GWAS, the correlation of effect sizes was r = 0.83 (P<0.0001) and the correlation of risk allele frequencies was r = 0.98 (P<0.0001). Conversely, for the 14 SNPs that had not previously reached genome-wide significance, the respective correlation coefficients were r = 0.24 (P = 0.43) and r = 0.72 (P = 0.003).

Univariable analysis using a multiplicative model revealed 8 SNPs that were nominally statistically significantly associated with melanoma at a P = 0.05 level (Table 2). All with the exception of rs1805005 had previously reached genome-wide significance in Melgene or GWAS. The strongest associations were observed for rs401681-T in locus *CLPTM1L* (OR 1.60, 95% CI 1.22–2.10; P = 0.001), rs16891982-C in locus *SCL45A2* (OR 0.51, 95% CI 0.34–0.76; P = 0.001), and rs1805007-T in locus *MC1R* (OR 4.38, 95% CI 2.03–9.43; P = 2×10^−5^). These 3 variants would also withstand multiple-testing Bonferroni correction for n = 33. The remaining five significantly associated variants were rs258322-T in *CDK10* (OR 2.26, 95% CI 1.32–3.88; P = 0.003), rs1805005-T in *MC1R* (OR 1.59, 95% CI 1.09–2.32; P = 0.016), rs1885120-C in *MYH7B* (OR 2.22, 95% CI 1.01–4.88; P = 0.047), rs2218220-T in *MTAP* (OR 0.74, 95% CI 0.56–0.97; P = 0.032) and rs4911442-G in *ASIP* region (OR 1.79, 95% CI 1.02–3.14; P = 0.042).

**Table 2:** Results of the univariable and multivariable analyses adjusting for hair color, skin color, eye color, phototype, sunburn and tanning and comparison with data from MelGene [23].

			Univariable analysis		Multivariable analysis		MelGene^2^		Known associations
SNP- Minor Allele	MAF	Gene Locus	OR (95% CI)	*P*-value	OR (95% CI)	*P*-value	OR (95% CI)	*p-value*	*Nevi/Pigmentation*
rs258322-T^1^	0.05	*CDK10*	2.26 (1.32–3.88)	0.003	1.77 (0.68–4.62)	0.241	1.66 (1.48–1.86)	4×10^−18^	No/Yes
rs401681-T	0.40	*CLPTM1L*	1.60 (1.22–2.10)	0.001	1.99 (1.21–3.26)	0.006	1.15 (1.08–1.22)	9.6×10^−6^	Weak/No
rs1805005-T	0.13	*MC1R*	1.59 (1.09–2.32)	0.016	1.61 (0.81–3.20)	0.179	1.13 (1.02–1.26)	0.024	No/Yes
rs1805007-T	0.02	*MC1R*	4.38 (2.03–9.43)	0.00002	5.50 (1.37–22.15)	0.016	1.83 (1.56–2.15)	2.7×10^−13^	No/Yes
rs1885120-C	0.02	*MYH7B*	2.22 (1.01–4.88)	0.047	3.10 (0.89–10.82)	0.0176	1.59 (1.41–1.79)	7.4×10^−15^	No/Yes
rs2218220-T	0.41	*MTAP*	0.74 (0.56–0.97)	0.032	0.54 (0.33–0.90)	0.05	0.84 (0.80–0.89)	5.5×10^−11^	Yes/No
rs4911442-G^3^	0.05	*(NCOA6) ASIP region*	1.79 (1.02–3.14)	0.042	3.29 (1.21–8.93)	0.02	1.2 (0.99–1.46)	1.03×10^−8^	No/Yes
rs16891982-C	0.14	*SCL45A2*	0.51 (0.34–0.76)	0.001	0.39 (0.17–0.89)	0.042	0.40 (0.33–0.47)	4×10^−27^	No/Yes

1 Association analysis on negative strand.

Abbreviations: NS, not significant.

2 MelGene status = Data from MelGene, an online database of reported genetic associations of melanoma including a systematic meta-analysis of melanoma-associated variants from published datasets and grading of these associations for strength of epidemiogical evidence [23]. OR (95% CI) and p value correspond to nominal association with melanoma after meta-analysis of data for each variant.

3 For this variant no meta-analysis was performed in MelGene due to the lack of sufficient datasets. The data represent those derived from the initial GWAS reporting an association of this variant with melanoma [50].

Five SNPs were significantly associated with CM in the multivariable analyses after controlling also for hair color, skin color, eye color, phototype, sunburn and tanning (Table 2).

### Power Considerations

The power of our study to detect ORs similar to those previously found, given the allele frequencies observed in the Greek population, ranges from 5.2% for rs12203592 to 100% for rs16891982 at α = 0.05. By summing the power estimates for all SNPs to detect the respective ORs seen previously, we estimated that if ORs were identical in the Greek population, our study would be expected to have found 8 nominally statistically significant associations among the 33 tested. Among the 18 variants that had been previously identified with genome-wide significance and did not show deviation from HWE, our study would be expected to have found 6 nominally statistically significant associations and 7 were indeed nominally significant.

### Comparison of risk allele frequencies between Greek sample and HapMap CEU

**Figure 2:**
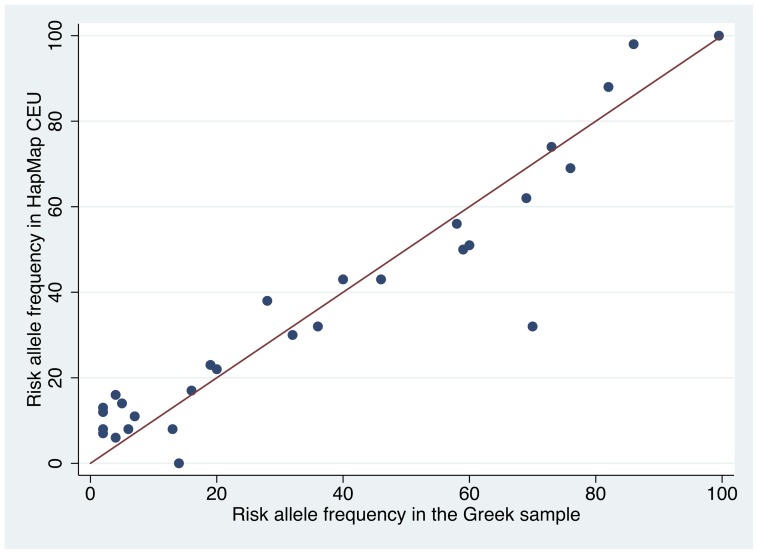
Correlation of risk allele frequencies. Correlation of the risk allele frequencies found in the Greek population and the frequencies of the same alleles from the HapMap CEU sample. Not shown are: rs4636294 (excluded from analyses because of HWE deviation); rs1805009 and rs11547464 because all subjects were homozygous for the respective major alleles and OR and hence risk allele could not be identified.

For 20 SNPs, the respective minor alleles were the risk alleles for melanoma. Table 3 shows risk alleles in the Greek sample and their frequency in both the Greek sample and HapMap CEU. The risk alleles in the Greek population had a median frequency of 20% (IQR, 4–60%), while their median frequency in HapMap CEU was 32% (IQR, 12–62%) (P = 0.243 for Mann-Whitney U). The correlation between the two populations was very high (r = 0.95, P<0.0001) (Fig. 2).

**Table 3:** List of genotyped SNPs, risk alleles in the Greek sample, and risk allele frequency in the Greek sample and HapMap CEU.

SNP	Risk allele in the Greek sample	Risk allele frequency in the Greek sample (95% CI)	Risk allele frequency in HaPMap CEU(95% CI)
rs16891982	G	0.86(0.83–0.89)	0.98(0.94–0.99)
rs401681	T	0.40(0.36–0.44)	0.43(0.36–0.50)
rs12203592	T	0.04(0.03–0.07)	0.16(0.11–0.21)
rs7023329	A	0.60(0.56–0.64)	0.51(0.44–0.57)
rs11515	C	0.82(0.79–0.85)	0.88(0.80–0.92)
rs3088440	A	0.07 (0.05–0.09)	0.11(0.07–0,15)
rs4636294^1^	N/A	N/A	0.50(0.43–0.57)
rs1335510	T	0.69 (0.65–0.73)	0.62(0.54–0.67)
rs2218220	C	0.59 (0.54–0.63)	0.50(0.43.0.57)
rs10757257	G	0.69(0.65–0.73)	0.62(0.55–0.68)
rs1011970	T	0.16 (0.13–0.2)	0.17(0.12–0.23)
rs1408799	T	0.32 (0.28–0.36)	0.30(0.24–0.36)
rs1042602	A	0.46 (0.42–0.51)	0.43(0.36–0.49)
rs1126809	A	0.20 (0.16–0.23)	0.22(0.14–0.29)
rs1393350	A	0.19 (0.16–0.23)	0.23(0.17–0.28)
rs1544410	G	0.58(0.54–0.62)	0.56(0.49–0.62)
rs1800407	A	0.06(0.04–0.08)	0.08(0.04–0.11)
rs258322	T	0.05(0.03–0.07)	0.14(0.09–0.18)
rs1805005	T	0.13(0.11–0.16)	0.08(0.04–0.12)
rs1805007	T	0.02(0.01–0.03)	0.12(0.08–0.17)
rs1805008	T	0.02(0.01–0.04)	0.13(0.08–0.17)
rs1805009	G	N/A	1 (NA)
rs1805006	C	0.995(0.98–0.999)	1(NA)
rs11547464	G	N/A	1(NA)
rs4785763	A	0.28(0.24–0.32)	0.38(0.31–0.44)
rs6058017	G	0.14(0.12–0.18)	0(NA)
rs4911414	G	0.76(0.72–0.79)	0.69(0.62–0.74)
rs1015362	G	0.73(0.69–0.76)	0.74(0.67–0.79)
rs910873	A	0.02(0.01–0.03)	0.08(0.05–0.12)
rs17305573	C	0.02(0.01–0.03)	0.08(0.05–0.12)
rs1885120	C	0.02(0.01–0.03)	0.07(0.04–0.11)
rs4911442	G	0.04(0.03–0.07)	0.06(0.02–0.12)
rs2284063	G	0.36(0.32–0.40)	0.32(0.25–0.38)
rs6001027	C	0.70(0.66–0.73)	0.32(0.25–0.38)

N/A: not applicable because all subjects were found homozygous for the major alleles.

1 Deviation from HWE, excluded from analyses.

The risk allele frequencies of nine SNPs (rs6001027-C, rs16891982-G, rs12203592-T, rs258322-T, rs1805007-T, rs1805008-T, rs910873-A, rs17305573-C, and rs1885120-C) were different beyond chance between the Greek sample and HapMap CEU (i.e. 95% CI of risk allele frequencies in the Greek population and the HapMap sample did not overlap). All these variants (except for rs6001027, a nevi-related SNP in *PLA2G6*) had significantly lower frequencies of risk alleles in the Greek population compared to HapMap CEU, while six of those are variants of genes with well-established role in the genetic control of pigmentation (rs16891982 in *SCL45A2*, rs12203592 in *IRF4*, rs258322 in *CDK10*, rs1885120 in *MYH7B*, rs1805007 and rs1805008 both in *MC1R*).

### Predictive value of predisposing SNPs in melanoma-associated risk factor models

**Figure 3:**
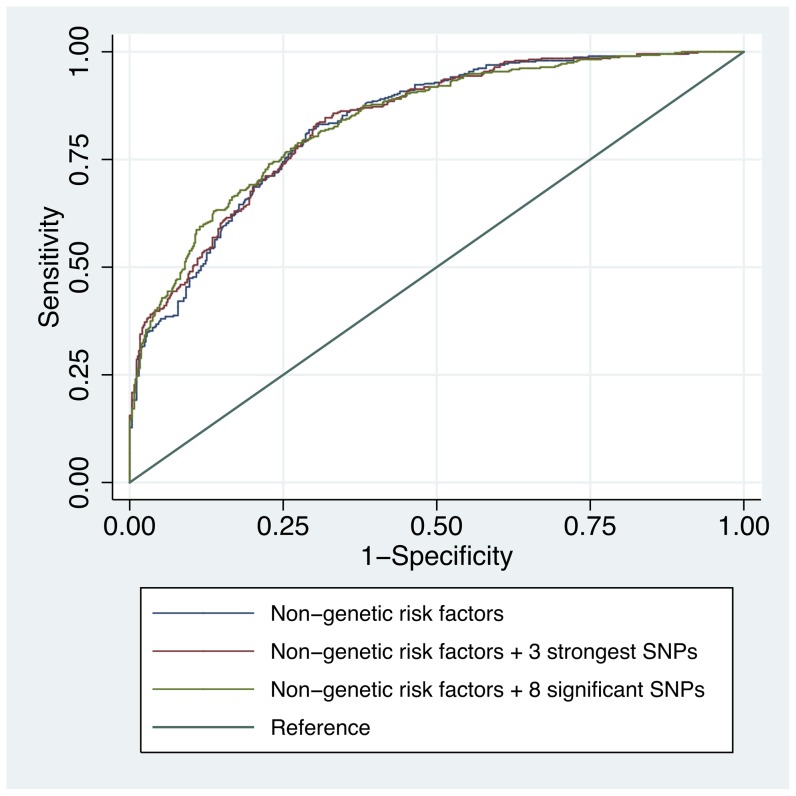
Areas under the curve (AUC) for 3 predictive models. Receiver operating characteristic curves for the 3 models including respectively the non-genetic risk factors alone, the non-genetic risk factors with the 3 SNPs surviving Bonferoni correction, and the non-genetic risk factors with all 8 nominally significant SNPs.

Figure 3 shows the areas under the curve (AUC) for 3 models considering different levels of genetic information. Compared to the phenotypic traits alone, models including the CM-associated SNPs only slightly improved the AUC. The AUC for the model that included only the nominally significant phenotypic traits (i.e. eye color, skin color, sunburn, phototype and tanning) (model 1) was 83.3%, whereas for the model that included these traits along with the 3 SNPs that remained significant after Bonferroni correction in the univariable analysis (model 2) was 83.7%, and the AUC for the model including the traits and all 8 SNPs with nominal significance (model 3) was 83.9%. Compared to the baseline non-genetic model, the genetic models did not confer a nominally significant improvement to the prediction of CM (P = 0.42 for model 1 vs. model 2, and P = 0.66 for model 1 vs. model 3).

## Discussion

We have replicated SNP-melanoma associations, with MAFs ranging from 2% to 41%. Eight associations were nominally statistically significant in the Greek population, the majority of which (87%) had previously reached genome wide significance. The replication of variants deriving from GWAS-discovered loci in our cohort, such as 20q11.2 (*ASIP* region), 9p21 (*MTAP* region), 16q24 (*MC1R* region) and 5p13 (*CLPTM1L region*), underscores the important contribution of the agnostic approach of GWAS in revealing genuine associations of genetic factors in complex diseases. For 8 SNPs the risk alleles had significantly lower frequencies in the Greek population compared to the HapMap CEU sample, while for 1 SNP the risk allele in the Greek population was higher than HapMap. The genetic models containing the SNPs that confer risk for melanoma improved the AUC compared with the model including only the phenotypic risk factors, but the improvement was of small magnitude.

The aim of our study was to validate a selected panel of SNPs in a case-control cohort of Greek descent, given our recent findings of a higher than expected genetic contribution of *CDKN2A/CDK4* mutations in a sizable cohort of sporadic and familial cases of our population [28]. Recent GWAS employing a higher density SNP tagging in large patient datasets has revealed a number of variants in genes involved in cell cycle regulation, telomere maintenance and DNA damage response, such as *MITF*, *ATM*, *PARP-1*, *TERT*, *CASP8*, *CCND1*, as well as polymorphisms in *MX2*, *SETDB1* and *ARNT/LASS2/ANXA9* region [31]–[34]. Although this study was based on earlier GWAS findings and certain candidate gene studies, our findings underscore the role of genes controlling pigmentary traits and DNA damage response in melanoma susceptibility in our population. This may reflect the importance of these pathways in melanoma development in a darker-skin population residing at an area of high year-round UV-influx. Most of the SNPs with significantly lower risk allele frequencies compared to HapMap CEU are found in loci implicated in pigmentation (*SCL45A2*, *IRF4*, *CDK10*, *MYH7B*, *MC1R*) and all but 2 (rs16891982, rs258322, rs1805007, and rs1885120) were replicated in the Greek population according to univariable analysis. These findings imply that there might be some differences in the genetic background underlying the phenotypical differences between the Greek and other European populations, and could partially explain the lower melanoma incidence in a population of darker skin complexion residing in a country with intense year-round UV exposure. In addition, our results may underscore the role of natural selection which tends to eliminate the prevalence of predisposing alleles in a population with high sun exposure and increase the frequency of protective alleles which also act through the protective pathways of pigmentation, However, Greeks harboring certain pigmentation-related risk alleles are at risk of developing melanoma.

In the case of melanocytic nevi, the comparison of allele frequencies between nevi-related variants in our cohort and the HapMap were less conclusive, with one variant (rs6001027) showing a higher allele frequency in our population. Only one (rs2218220 in the MTAP region, chrom. 9p21) of the previously nevi-associated SNPs was found to be positively associated with melanoma in our analysis. Given that nevi have been shown to be a strong risk factor of melanoma in the Greek population [35], it is likely that our study was not powered enough to detect smaller effect sizes conferred by these variants. In addition, other nevus-associated variants, yet uncovered, may play a role in melanoma risk.

Among the three top variants of our analysis, the most prominent locus was located within the cleft lip and palate transmembrane 1-like (*CLPTM1L*) gene and the telomerase reverse transcriptase (*TERT*) gene. The major C allele of rs401681 has been repeatedly reported to confer risk for BCC and protection against melanoma [36]–[39], and was recently replicated at a GWAS of 2,981 melanoma patients and 1,982 controls [31]. In addition, a meta-analysis including data from an Australian case-control study showed that *TERT–CLPTM1L* variants do influence melanoma risk, albeit with a relatively small effect size [32]. The “red hair” variant rs1805007 of the *MC1R* gene has been consistently linked to melanoma risk in relevant studies. In meta-analyses, rs1805007 showed the highest attributable risk for melanoma among *MC1R* variants [13], [40] with effect estimates similar to those found in this study and a previous Greek case-control study [16]. rs16891982 of the *SLC45A2*, influences skin pigmentation and exhibits substantially different frequencies among populations, thus determined as an ancestry informative marker. The ancestral Leu allele (rs16891982-C) has been associated with dark skin, eye, and hair color in whites [41], while exhibiting a protective effect against melanoma [39], [42]–[44].

The variants selected for this study were based on the results of a large field synopsis and on-line database that scrutinized all published data on the genetic association of melanoma and subjected them to systematic meta-analyses. All but one (rs1805005) nominally significant associations in our selected set of SNPs came from a subgroup of variants which had p values of 10^−7^ and are likely to represent genuine associations [45]. We were also able to assess the predictive value of genetic factors in models incorporating various phenotypical and genetic risk factors. In the examined models, the predictive value of AUC did not substantially improve by the addition of genetic variants, compared with the model that involved only the clinical risk factors. Although these genetic models do not seem to contribute substantially to melanoma risk prediction, they are nevertheless suggestive of the contribution of low-penetrance gene variants to melanoma risk. Failure of models relying on common gene variants to improve substantially the predictive discrimination of traditional risk factors is a common problem encountered in complex diseases. Much larger effect sizes and a very large number of genetic variants are needed to improve perceptively the predictive value of genetic models [46]. Moreover, our findings show that statistical significance of a risk model does not guarantee clinical utility highlighting the distinction between the statistical and clinical perspectives of genetic risk models [47].

The current study has some limitations. First, the sample size is modest resulting probably in limited power to detect small or even moderate effects for additional SNPs. Second, no data were recorded on the number of nevi, a well-known melanoma risk factor for melanoma. Nevertheless, only one (rs2218220 in *MTAP*) of the eight SNPs associated with melanoma has been reported to be also associated with nevus count [48]. It is possible that rs2218220 would lose its significance as a melanoma-associated variant if the number of nevi were included in the multivariate analyses. Third, failure to replicate candidate loci in pigmentation-associated genes other than *MC1R*, *SLC45A2*, *CDK10*, *MYH7B and ASIP region* could derive from a lack of sufficient statistical power. Fourth, we selected our SNPs from the last update (October 2011) of the MelGene database. However, in the meantime between updates new SNPs might have been discovered in new GWAS, which are likely not to have been included in the accumulated evidence reported in MelGene because of the practical issues of intervals between database updates. This limitation may have a limited impact since some of the newest GWAS, which are not included in this paper, i.e., Barrett et al 2011 [31], provide estimates for established genetic risk factors on expanded datasets of previous GWAS, i.e., Bishop et al 2009 [49], the results of which are included in this study.

In conclusion, our research validated a number of variants that contribute to melanoma susceptibility in Greek population. The assessment of genetic input in a population with one of the lowest incidence of the disease could highlight the variation of genetic risk factors that are in-play in different environmental and population settings from those used in the majority of previous studies. Further validation of newly described variants and a better understanding of the gene-environment interaction may provide valuable insight in the variation of melanoma risk among white populations of different ancestry.

## Supporting Information

Table S1 **Demographic characteristics and pigmentary phenotype of melanoma cases and control subjects.**
(DOCX)
