# Supplementary material for: Replication and Predictive Value of SNPs Associated with Melanoma and Pigmentation Traits in a Southern European Case-Control Study
Source: PLoS One. 2013 Feb 5;8(2):e55712. doi: 10.1371/journal.pone.0055712 (PMC3564929; doi:10.1371/journal.pone.0055712)
Supplement: Table S1 — Demographic characteristics and pigmentary phenotype of melanoma cases and control subjects. (DOCX) [file pone.0055712.s001.docx]

**Table S1. Demographic characteristics and pigmentary phenotype of melanoma cases and control subjects.**

|  | **Patients (n=284)^1^** | **Controls (n=284)^1^** | ***P*-value** |
| --- | --- | --- | --- |
| **Median age** (years) (IQR; range) | 44 (35-54; 18-85) | 42 (31-52; 18-81) |  |
| **Men**, N (%) | 135 (48%) | 135 (48%) |  |
| **Hair color** |  |  | 0.104 |
| Light Hair | 31 | 27 |  |
| Dark Hair | 186 | 255 |  |
| **Eye color** |  |  | 0.073 |
| Light Eyes | 40 | 71 |  |
| Dark Eyes | 177 | 211 |  |
| **Skin color** |  |  | <0.0001 |
| Light Skin | 122 | 265 |  |
| Dark Skin | 95 | 17 |  |
| **Phototype** |  |  | <0.001 |
| Phototype I | 9 | 12 |  |
| Phototype II | 109 | 69 |  |
| Phototype III | 75 | 141 |  |
| Phototype IV | 23 | 55 |  |
| **Tanning ability** |  |  | <0.0001 |
| Burn | 35 | 34 |  |
| Minimal tan | 98 | 66 |  |
| Burn then tan | 70 | 104 |  |
| Deep tan | 12 | 73 |  |
| **Lifetime sunburn** |  |  | 0.301 |
| Sunburn – yes | 91 | 136 |  |
| Sunburn – no | 115 | 142 |  |

^1^ The sum of the numbers of subjects in some of the strata was fewer than the total number of subjects because some subjects did not provide the information. Missing values were imputed using multiple imputation methods.

Abbreviations: IQR, interquartile range
